# No Evidence for a Global Male-Specific Lethal Complex-Mediated Dosage Compensation Contribution to the Demasculinization of the *Drosophila melanogaster X* Chromosome

**DOI:** 10.1371/journal.pone.0103659

**Published:** 2014-08-05

**Authors:** Steven P. Vensko, Eric A. Stone

**Affiliations:** 1 Program in Genetics, North Carolina State University, Raleigh, North Carolina, United States of America; 2 Department of Biological Sciences, North Carolina State University, Raleigh, North Carolina, United States of America; CNRS, France

## Abstract

In *Drosophila melanogaster* males, the expression of *X*-linked genes is regulated by mechanisms that operate on a chromosomal scale. One such mechanism, male-specific lethal complex-dependent *X*-linked dosage compensation, is thought to broadly enhance the expression of male *X*-linked genes through two-fold transcriptional upregulation. The evolutionary consequences of this form of dosage compensation are not well understood, particularly with regard to genes more highly expressed in males. It has been observed the *X* chromosome arrangement of these male-biased genes is non-random, consistent with what one might expect if there is a selective advantage for male-biased genes to avoid dosage compensation. Separately, it has been noted that the male-specific lethal complex and its dosage compensation mechanism appear absent in some male tissues, thus providing a control for the selection hypothesis. Here we utilized publicly available datasets to reassess the arrangement of *X*-linked male-biased expressed genes after accounting for expression in tissues not dosage compensated by the male-specific lethal complex. Our results do not corroborate previous observations supporting organismal-wide detrimental effects by dosage compensation on *X*-linked male-biased expressed genes. We instead find no evidence that dosage compensation has played a role in the arrangement of dosage compensated male-biased genes on the *X* chromosome.

## Introduction

Phenotypic contrasts between *Drosophila melanogaster* males and females have revealed a vast number of traits to be sexually dimorphic. Technological advances have enabled similar contrasts of molecular endophenotypes, and again sexual dimorphism has been shown to be widespread, especially within the transcriptome [1]–[3]. A number of studies have sought to understand the role and evolutionary history of *Drosophila* sex-biased expressed genes, with an emphasis on *X*-linked male-biased expressed genes (MBGs) [4]–[8]. Fundamental to these is an understanding of the sex-specific molecular mechanisms that globally regulate *X*-linked gene expression.

In *Drosophila*, two primary mechanisms are thought to broadly modulate *X*-linked gene expression in males: meiotic sex chromosome inactivation (MSCI) [9], [10] and male-specific lethal (MSL) complex-mediated dosage compensation (referred hereafter simply as dosage compensation or DC) [11]. During MSCI, the male *X* chromosome is rendered inactive in meiotic tissue; in DC, transcriptional activity on the male *X* chromosome is generally upregulated in somatic tissues in order to compensate for male *X* monosomy. The extent to which these operate remains a subject of debate [12]–[14] as do their evolutionary implications [5], [8], [15]. It has been argued DC is selectively disadvanteous for some somatically expressed *X*-linked MBGs; likewise, many meiotically expressed *X*-linked MBGs are thought to be negatively impacted by MCSI. Evidence supporting these hypotheses is indirect. First, throughout the Drosophila genus, there appears to be a deficit of both meiotically and mitotically expressed MBGs on the *X* chromosome [4]. Secondly, sequence comparisons have revealed an enrichment of *X* 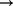 Autosome retrotransposition events affecting both classes of MBGs [16], [17]. Third, an association has been observed between the degree to which an MBG is male-biased and how far the gene resides from the nearest chromatin entry site (CES) in gonads, gonadectomized flies and whole flies [18]; this observation is relevant to DC because CESs are the primary binding targets for the MSL (*Male-specific Lethal*) complex, whose role in dosage compensation is discussed below [19], [20].

*Drosophila* dosage compensation (reviewed in [21]) involves the upregulation of the heterogametic *X* chromosome in male somatic tissues [11]. Dosage compensation is mediated by a ribonucleic complex known as the MSL complex which consists of at least five separate components (*MSL-1 (male-specific lethal 1*), *MSL-2 (male-specific lethal 2)*, *MSL-3 (male-specific lethal 3)*, *MOF (male absent on the first)* and *MLE (maleless)*) as well as two redundant yet unique non-coding RNAs (*roX1* and *roX2*). The histone modification associated with dosage compensation, H4K16Ac [22] is suspected of allowing increased accessibility of transcription factors to regulatory regions [23], [24] as well as aiding in hypertranscription through augmented transcriptional elongation [25].

This manuscript focuses on DC in *Drosophila* and its putative impact on the organization of the *X* chromosome. The prevailing argument for how DC has influenced the *X* hypothesizes selection against MBGs that are both dosage compensated and further upregulated by non-DC mechanisms [18]. Under certain assumptions, this selection would favor the migration of MBGs away from CESs, including retrotranspositions to the autosomes. As mentioned above, Drosophila *X* chromosomes carry a signature conistent with such migrations; however, other inconsistencies persist. There remains debate as to whether the *X* chromosome is truly depauperate for MBGs, with some suggesting the observation is confounded by the absence of DC in the testis [8]. Among those who accept that MBGs are depleted on the *X* chromsome, there remains debate as to why. The arguments in the literature have been fueled by analyses utilizing increasingly rich datasets that have yielded seemingly contradictory results [15], [26]. To help rectify these apparent contradictions, we tested for incompatibility between dosage compensation and elevated transcriptional activity as well as reassessed evidence suggesting DC shapes the arrangement of MBGs on the *X* chromosome, explicitly controlling for the absence of any MSL complex-like dosage compensation in meiotic tissues [27], [28]. Our approach complements the previous dissection-based approach by Bachtrog and colleagues [18]. We are specifically interested in testing whether the high male-bias magnitudes from tissues that are not dosage compensated by the MSL complex (or any MSL complex-like mechanism) drive the signal for highly male-biased genes being distant from chromatin entry sites in whole flies. The Results section that follows is organized around each of these reanalyses.

## Results

### No evidence for incompatibility between elevated somatic *X*-linked MBGs transcript abundance and its probability of dosage compensation

**Figure 1:**
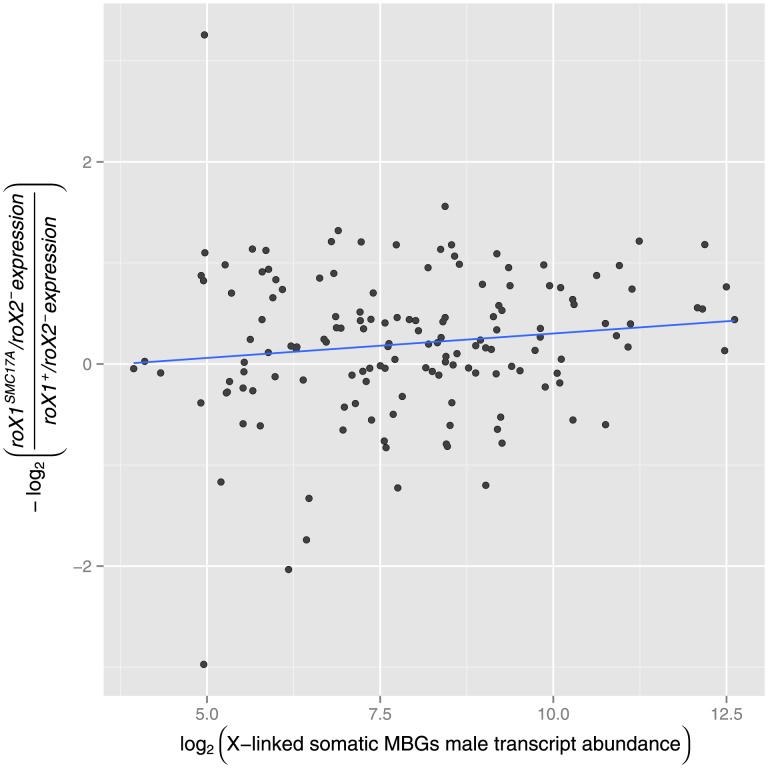
Degree of dosage compensation versus transcript abundance. For *X* -linked MBGs, a measure of dosage compensation (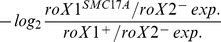) is plotted against and transcript abundance in third instar larval males. No significant linear relationship was found (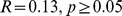).

Previous work identified a depletion of *X*-linked MBGs near CESs and found the degree of male bias to be greater for MBGs more distant from their nearest CES [18]. These observations were interpreted as the signature of far-reaching selection driven by an unfavorable interaction between DC and extensive upregulation by non-DC mechanisms. Specifically, it has been argued that such hypertranscription in males may result in a mechanistic or functional limitation on transcriptional activity [7], [18]. If these hypotheses were true, one might expect reduced dosage compensation for genes already highly expressed, in particular for those genes already male biased. Experiments that abolish DC allow these expectations to be tested. We made use of a dataset generated by comparing wild-type transcript abundance to the abundance measured in mutants whose MSL complex was disrupted [29]. Specfically, the experiment utilized a severe *roX1* mutant (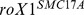) to determine the *roX*'s role in localizing the MSL complex to the *X* chromosome. Expression was measured in both the mutant 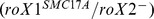 and wild-type 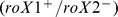 third instar larval males to quantify the degree to which *X*-linked transcriptional activity was reduced. Despite not rendering the MSL complex completely inactive, Deng and Meller [29] found expression, on average, was reduced to levels similar to an *MSL-2* RNAi treatment. We chose to use this dataset over *MSL-2* RNAi datasets (see [11], [30]) due to the increased number of expressed genes [29] and increased similarity to adult males relative to S2 cell lines. We used the results of this experiment to test for an association between wild-type transcript abundance and the negative log ratio of mutant to wild-type transcript abundance for expressed somatic *X*-linked MBGs. No such association was found (Figure 1, 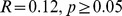). Expectedly, *X*-linked female-biased and unbiased genes also show no evidence of an incompatibility of elevated transcriptional activity and dosage compensation (see Figure S1). In summary, we found no evidence of a detrimental relationship among *X*-linked genes, male biased or otherwise, between the magnitude of transcript abundance and the degree of dosage compensation. This result is supported by observations by Meisel and colleages [15] who were unable to find a depletion of larval expressed male-biased expressed genes on the *X* chromosome. Together, this suggests any detrimental effects by DC is either stage-specific or, more likely, applicable to specific tissues that may not be present in third instar larval males. We next sought evidence instead in the spatial distribution of MBGs on the *X* chromosome.

### No evidence for dosage compensation driving selection favoring more male-biased somatic *X*-linked MBGs further from chromatin entry sites

**Figure 2:**
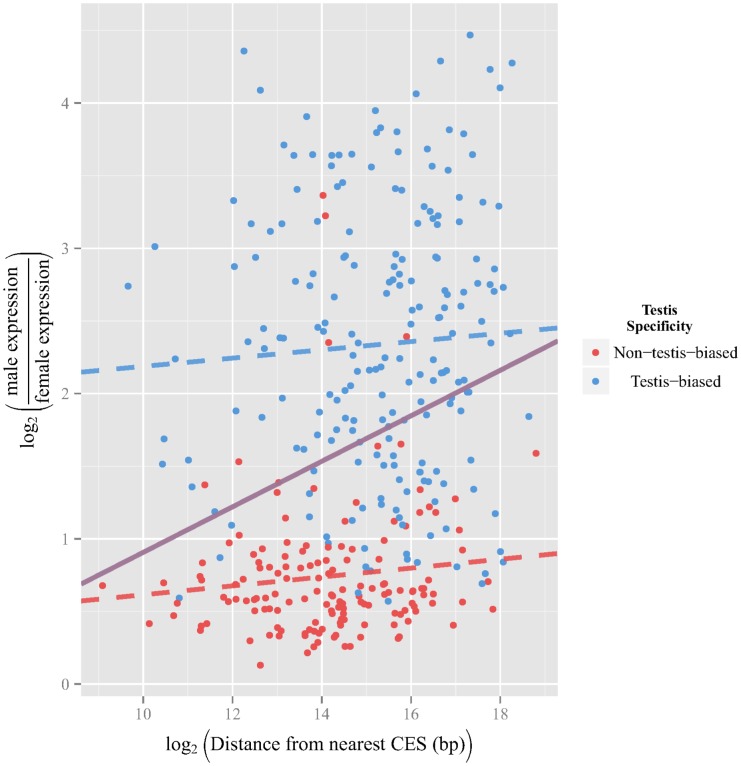
Degree of male expression bias versus distance from nearest chromatin entry site. The degree of male expression (as measured by 
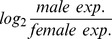
) for each gene is plotted against the logarithmic distance to its nearest chromatin entry site. We recapitulate the previous observation of a significant positive correlation (purple line, 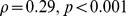); however, after partitioning *X*-linked MBGs into testis-biased and non-testis-biased the relationship between male-bias magnitude and distance from the nearest CES becomes non-significnat for non-testis-biased *X*-linked MBGs (red dashed line, 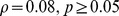) and testis-biased genes (blue dashed line, 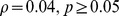).

Recall that *X*-linked MBGs have been observed to be further from their nearest CES than *X*-linked FBGs or UBGs and that greater degrees of male-biased expression have been observed among *X*-linked MBGs that are relatively far from their nearest CES. [18]. In our reanalysis, we used data from SEBIDA [31] and quantified the degree of sex-bias as the difference between male and female expression after 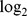 transformation. For each *X*-linked MBG gene, we plotted in Figure 2 its degree of male-bias against the distance to its nearest CES (from [19]); the latter values were also 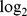-transformed, though this was done primarily to aid visualization. As indicated in purple, there is a significant positive correlation (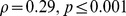), suggesting that, when all *X*-linked MBGs are considered, the degree of male bias does increase with as distance from the nearest CES increases. Under the assumptions that DC diminishes away from CESs and that DC may be detrimental to strongly male-biased genes, observing an increased bias away from CESs may be indicative of historical selection. Since genes expressed primarily in the testis are not dosage compensated by the MSL complex and are often male biased, the observed association might result if such genes tend to be distant from their nearest CES. Indeed, testis-biased MBGs tend to be significantly further from their nearest CES than somatically expressed MBGs (Figure S2, somatically expressed MBG mean distance from nearest CES  = 34,980 bp, testis-biased MBG mean distance from nearest CES  = 72,908 bp, 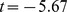, 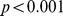). To test for the contribution of non-dosage compensated testis-biased MBGs to the association signal we partitioned MBGs into a testis-biased MBGs set and somatically expressed MBGs set. Upon doing so, we find no marginal relationship between degree of male bias and proximity to the nearest CES for somatically expressed MBGs (Figure 2, dashed red line, 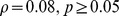) or for testis-biased MBGs (Figure 2, dashed blue line, 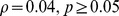). This pattern was also observed using expression data from a variety of experiments (see Figure S3, Figure S4, Figure S5, Figure S6, Figure S7, and Figure S8).

**Figure 3:**
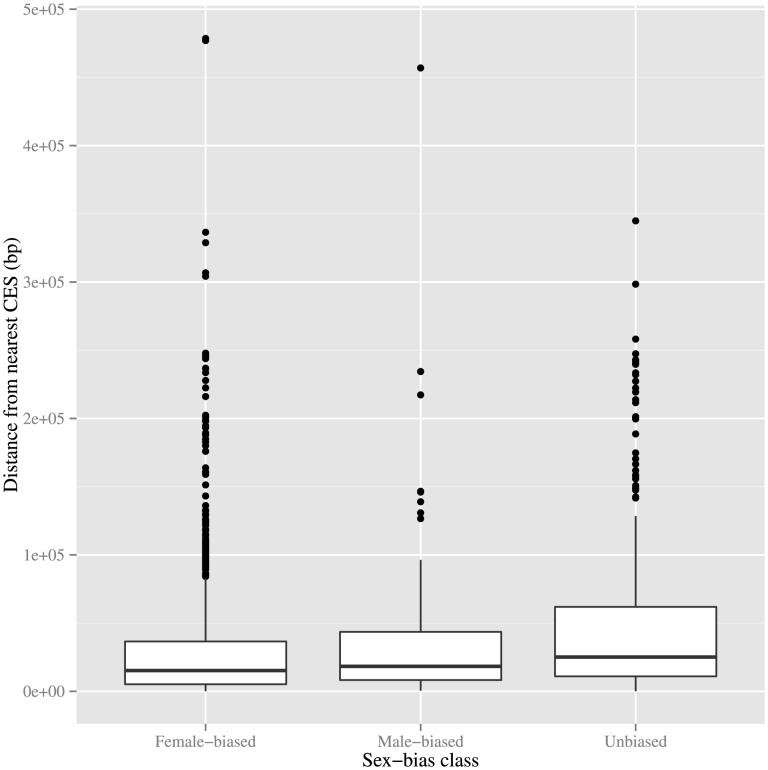
Distances of *X*-linked, non-testis-biased genes from nearest chromatin entry site, stratified by sex-bias class. Each *X*-linked gene, excluding those whose expression is biased towards testis, is classified by its expression pattern as female-biased (left), male-biased (middle), or unbiased (right). The boxplots represent the distances of genes within each class from their nearest chromatin entry site. No significant difference in mean distance is observed between the male-biased and female-biased classes. Interestingly, the male-biased genes appear to be significantly closer to the nearest CES than their unbiased counterparts (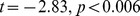).

Another observation supporting a hypothesis of detrimental effects by dosage compensation on *X*-linked MBGs is *X*-linked MBGs tending to be, on average, further from their nearest CES than *X*-linked FBGs or UBGs. We were unable to find any significant difference in distance from the nearest CES between somatically expressed *X*-linked MBGs and *X*-linked FBGs (Figure 3, 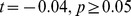). Once again, this suggests non-dosage compensated testis-biased expressed MBGs may have been skewing the distribution of distances from nearest CES for *X*-linked MBGs. Unexpectedly, we found *X*-linked somatically expressed MBGs tend to be closer to their nearest CES than *X*-linked UBGs are to their own closest CES (Figure 3, 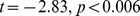). These results does not exclude the role of DC in shaping the spatial distribution of MBGs relative to CESs; however, because DC is not present in testis, if such a role exists then it does not appear to act on an organismal scale.

## Discussion

Our study is meant to complement previous work in which Bachtrog and colleagues [18] observed a significant relationship between increased male-bias magnitude and distance from the nearest CES in whole flies, gonads and gonadectomized males. We were able to recapitulate the previous observation of a correlation between *X*-linked male-bias magnitude and distance from the nearest CES using magnitude measurements from a variety of experiments (see Supporting Information). Concerned that the increased distance of non-dosage compensated testis-biased MBGs from their nearest CES (and the signal observed by Bachtrog and colleagues), we filtered these genes from our analysis and in doing so abolished any relationship among MBGs between CES proximity and either transcript abundance or degree of male bias. Thus, it is possible that this spatial bias was the driver behind previous observations. It is also noteworthy that Bachtrog and colleagues [18] examined transcript abundance in gonadectomized flies from Parisi et al. [2] and found *X*-linked MBGs to be significantly further from their nearest chromatin entry site than either *X*-linked FBGs and UBGs. They moreover found the degree of male bias to increase with distance from the nearest chromatin entry site. On the surface, these observations would seem to contradict our attribution of whole-body signal to germline tissue; however, we suggest an explanation that rectifies our conclusions with those of Bachtrog et al [18]. The observations by Bachtrog and colleagues are consistent with a residual signal from testis-biased genes. Such a signal would obviously persist if the excision of germline tissue was incomplete; more subtly, the same would be seen if testis-biased genes are detectably expressed and male biased outside of the germline. In an attempt to remove any residual signal from testis-biased genes, our analyses excluded the (somatic) expression data of testis-biased genes from our somatic MBG set. When applying our approach to the gonadectomized data used by Bachtrog and colleagues, we detected a significant association between sex-bias magnitude and distance from the nearest CES for MBGs. Testing for this association using only somatically expressed MBGs or only testis-biased MBGs resulted in no significant association for either set (See Figure S8). These results are consistent with the hypothesis that testis-biased MBGs are expressed and male biased in the gonadectomized carcass data, either due to incomplete excision or more likely somatic expression.

Other results not considered here have been used as evidence that DC may negatively impact *X*-linked MBGs. For example, differential MSL-complex binding patterns were shown to exist among various relevant classes of *X*-linked genes (see [18] using data from [32]). Though the results are potentially of interest, we chose not to reassess the underlying analyses due to the nature of the binding data (also noted in [8]). The data employed [32] come from “male-like” cell lines which express primarily housekeeping genes. Because the MSL complex is known to preferentially bind actively expressed genes [32]–[34], and because the assayed cell lines and embryos may have drastically different expression patterns than third instar larval males (see [29]) and presumably adult males, one might not expect the MSL complex to often bind to MBGs. Additionally, MSL-complex binding is an imperfect surrogate for being dosage compensated. DC has been observed among genes not directly bound by the MSL complex [35] and H4K16Ac is distributed more broadly than is MSL binding [22]. This suggests that, even with ideal MSL-compex binding data, a gene classified as not bound by the MSL complex may very well be dosage compensated.

Although we found no evidence that DC has contributed globally to a depletion of *X*-linked MBGs, DC may indeed have detrimental effects in specific tissues. Meisel and colleagues [15] provide convincing evidence that DC and sexual conflict may have contributed to the depletion of *X*-linked MBGs expressed in the accessory gland. Disentangling the role of dosage compensation, if any, will likely be be difficult due to potential interactions with sexual conflict [36].

The extent to which DC negatively impacts *X*-linked MBGs remains unresolved, as do any potential evolutionary consequences. It does appear, as in the case of male accessory glands, that there may be selective consequences of dosage compensation [8], [15]. Our work suggests that, while such tissue-specific cases may exist, they do not contribute to any discernable of an organismal-wide pattern. In doing so, we demonstrated the quantitative effects of assuming DC to act uniformly across tissue types. Our treatment, inspired by Meiklejohn and Presgraves [8], was digital in that we considered only somatically expressed MBGs and thus controlled for the lack of dosage compensation in the testis. A more sensitive approach would consider the extent of dosage compensation on a tissue-by-tissue basis; while such data does not to our knowledge exist, we believe it would go a long way toward clarifying the role of DC in male *X*-linked gene expression and the evolutionary consequences thereof.

## Methods

Data were collected from their respective latest public release. Specifically, probe-set-based expression measurements from third instar larval males in both the 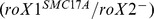 and 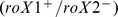 genetic backgrounds were collected from [29] (http://www.genetics.org/content/174/4/1859/suppl/DC1). Genes were partitioned into testis-biased expressed and somatically expressed using tissue-specific expression data from FlyAtlas [37]. Specifically, genes were classified as testis-biased if expression was highest in the adult testis for all four FlyAtlas replicates and if 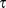 (see [38]) 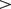
0.9. Sex-bias classifications and magnitudes were acquired from the Sex Bias Database [31] (SEBIDA) version 3.1 release. In particular, we used the “meta” sex-bias classifications and sex-bias magnitudes which utilize expression data from a variety of sources (including [1]–[3] among others). Genes were classified as *X*-linked or autosomal based on the FB2013_04 Release gene map table from FlyBase [39]; gene coordinates, used to determine gene distance from CESs, were also obtained from this source. CES locations (Release 5.5 coordinates) were obtained from the supplemental data from [19].

Custom Perl scripts were created (available upon request) to merge all of the collected data into a single table. Specifically, all genes, regardless of the identification system they were made available in, were converted to FBgn IDs using the FB2013_02 Release FBgn annotation file form Flybase [39]. To prevent bias, we excluded genes for which the mapping from older ID to updated ID was not unique. Probe sets were assigned to FBgn IDs using the Affymetrix Build 33 probe set annotation file. In the expression datasets we considered (e.g. [29]), genes were deemed expressed when called “present” in all replicates. In these cases, the replicates were averaged (as were the values across probe sets for genes spanning multiple ones) and assigned to the appropriate FBgn ID. Distance from a gene to its nearest CES was calculated as the minimum number of base pairs between the midpoint of the gene and the midpoint of any CES. The data, after filtering, included 1398 *X*-linked genes, 348 of which were male biased.

Custom R scripts (available upon request) using standard R functionality were created for statistical analysis. All 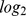 transformations of sex-bias magnitudes, expression ratios, and gene distances from CES occured within these scripts.

## Supporting Information

Figure S1
**Distances of** ***X*** **-linked testis-biased and non-testis-biased male-biased expressed genes from their nearest chromatin entry site.** *X*-linked MBGs were classified as testis-biased or non-testis-biased base on tissue specificity calculated using tissue-specific data from FlyAtlas. Testis-biased *X*-linked MBGs tend to be significantly further from their nearest CES (mean distance: 72909 bp) than non-testis-biased *X*-linked MBGs (mean distance: 34980 bp)(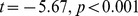).(EPS)

Figure S2
**Relationship between transcript abundnace and dosage compensation for** ***X*** **-linked FBGs and UBGs.** A combined set of *X*-linked FBGs and UBGs do not show any compatibility between transcript abundance and probability of dosage compensation. Interestingly there is instead a positive correlation suggesting that more highly transcribed genes show a stronger signature of dosage compensation (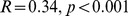). The authors hypothesize this is an artifact of the sensitivity of the arrays used.(EPS)

Figure S3
**Relationship between sex-bias magnitudes and distance from nearest CES using whole fly expression data from** ***Innocenti and Morrow, 2010*** **.** Using whole body expression from *Innocenti and Morrow, 2010* we found a significant positive correlation between sex-bias magnitude and distance from the nearest CES including all *X*-linked MBGs (purple line, 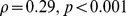). After partitioning into testis-biased and non-testis-biased we were unable to find any significant correlation for non-testis-biased MBGs (dashed red line, 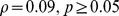) or testis-biased MBGs (dashed blue line, 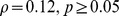).(EPS)

Figure S4
**Relationship between sex-bias magnitudes and distance from nearest CES using whole fly expression data from** ***Wyman et al., 2010*** **.** Using whole body expression from *Wyman et al., 2010* we found a significant positive correlation between sex-bias magnitude and distance from the nearest CES when considering all *X*-linked MBGs (purple line, 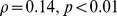). After partitioning *X*-linked MBGs into testis-biased and non-testis-biased we were unable to find any significant correlation for non-testis-biased MBGs (dashed red line, 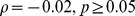) or testis-biased MBGs (dashed blue line, 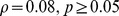).(EPS)

Figure S5
**Relationship between sex-bias magnitudes and distance from nearest CES using whole fly expression data from** ***Ayroles et al., 2009*** **.** Using whole body expression from *Ayroles et al., 2009* we found a significant positive correlation between sex-bias magnitude and distance from the nearest CES when considering all *X*-linked MBGs (purple line, 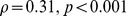). After partitioning *X*-linked MBGs into testis-biased and non-testis-biased we were unable to find any significant correlation for non-testis-biased MBGs (dashed red line, 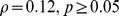) or testis-biased MBGs (dashed blue line, 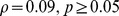).(EPS)

Figure S6
**Relationship between sex-bias magnitudes and distance from nearest CES using head-specific expression data from** ***Goldman et al., 2007*** **.** Using head-specific expression from *Goldman et al., 2007* we could not find a significant positive correlation between sex-bias magnitude and distance from the nearest CES when considering all *X*-linked MBGs (purple line, 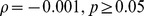). It is unnecessary to filter out testis-biased MBGS since somatic comparisons between males and females do not allow for the excessively high male-bias magnitudes from the germline to drive any artifactual signals.(EPS)

Figure S7
**Relationship between sex-bias magnitudes and distance from nearest CES using whole fly expression data from** ***Stolc et al., 2004*** **.** Using whole body expression from *Stolc et al., 2004* we found a significant positive correlation between sex-bias magnitude and distance from the nearest CES when considering all *X*-linked MBGs (purple line, 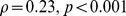). After partitioning *X*-linked MBGs into testis-biased and non-testis-biased we were unable to find any significant correlation for non-testis-biased MBGs (dashed red line, 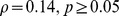) or testis-biased MBGs (dashed blue line, 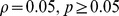).(EPS)

Figure S8
**Relationship between sex-bias magnitudes and distance from nearest CES using gonadectomized carcass expression data from** ***Parisi et al., 2004*** Using gonadectomized carcass expression from *Parisi et al., 2004* we found a significant positive correlation between sex-bias magnitude and distance from the nearest CES when considering all *X*-linked MBGs (purple line, 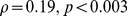). After partitioning *X*-linked MBGs into testis-biased and non-testis-biased we were unable to find any significant correlation for non-testis-biased MBGs (dashed red line, 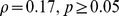) or testis-biased MBGs (dashed blue line, 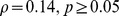). While we would not expect gonadectomzied carcasses to show extreme male-biased magnitudes since the germline has been removed, it is possible that other somatic tissues, such as the male accessory gland, may have contributed to this signal.(EPS)
